# Comparing the perceptions and opinions of the 2007 and 2019 Canada's food guides among parents of young children

**DOI:** 10.3389/fpubh.2022.944648

**Published:** 2022-08-09

**Authors:** Alyssa V. Ramuscak, David W. L. Ma, Laura E. Forbes, Alison M. Duncan, Adam Sadowski, Jess Haines

**Affiliations:** ^1^Department of Family Relations and Applied Nutrition, University of Guelph, Guelph, ON, Canada; ^2^Department of Human Health and Nutritional Sciences, University of Guelph, Guelph, ON, Canada

**Keywords:** food-based dietary guidelines, Canada's Food Guide, perceptions, opinions, parents

## Abstract

**Background:**

The Canada's Food Guide (CFG) is recognized as the most prominent authoritative guideline for healthy eating in Canada. In 2019, Health Canada released the latest iteration of the CFG with substantial changes to its messaging and format from the previous 2007 CFG.

**Objective:**

This study compared the awareness, use, knowledge, and opinions of the 2007 and 2019 CFGs among parents with children aged 18 months to 5 years who are participants in a family-based intervention trial, the Guelph Family Health Study.

**Methods:**

The sample consisted of 327 parents (59% women) who responded to questions about the 2007 CFG and 177 parents (60% women) who responded to questions about the 2019 CFG. Parents' awareness and knowledge of the 2007 and 2019 CFGs were compared using Pearson's Chi-Square, while parents' opinions of the two CFGs were compared using Wilcoxon Rank-Sum tests. To describe and provide context about how parents used the 2007 and 2019 CFG descriptive analysis was used. To analyze the open-answer comments parents provided for the 2007 and 2019 CFGs thematic coding was used.

**Results:**

Awareness of the 2007 and 2019 CFGs was high with 94.5 and 90.4% of parents reported having heard about the 2007 and 2019 CFGs, respectively. Knowledge of the plate proportion recommendations in the 2019 CFG was significantly higher than knowledge of the recommended number of servings in the 2007 CFG with 93.4% of parents identifying the Vegetable and Fruit Plate Proportions in the 2019 CFG. Parents identified that the 2019 CFG was a helpful and trustworthy resource, and that it was easier to follow and understand, and more representative of their culture and traditional foods than the 2007 CFG.

**Conclusion:**

Our results suggest that parents' knowledge of the 2019 CFG recommendations was higher than for the 2007 CFG recommendations. Parents also had more positive opinions about the 2019 CFG as compared to the 2007 CFG. Future research is needed to explore whether these higher levels knowledge of the 2019 CFG recommendation translate to healthier eating patterns among Canadian families.

## Introduction

The World Health Organization (WHO) describes healthy eating as the cornerstone to good health and nutrition (1). Consuming a healthy diet rich in plant-based foods such as vegetables, fruit, whole grains, legumes, lentils, and nuts and seeds, throughout the life course can support healthy growth and development, prevent malnutrition, and reduce the risk of developing chronic diseases like obesity, type 2 diabetes, and hypertension (2). However, there is evidence to suggest that many Canadian children and adults' diets are poor and fail to meet dietary recommendations (3, 4).

Of the many factors that can influence one's diet, nutrition knowledge has been described as the most amenable to change and has been the driver of numerous nutrition interventions and health campaigns (5, 6). Existing research suggests a weak, positive relationship between knowledge and consumption of healthy foods and dietary patterns among both adults and children (5, 7–17). These results underscore the idea that nutrition knowledge may be a necessary, although not sufficient, factor in facilitating and supporting healthy eating. Thus, it is vital that credible, evidence-based education, tools, and resources be available and accessible to inform Canadians' eating patterns and behaviors.

The Canada's Food Guide (CFG) is a knowledge translation tool that translates nutrient requirements and scientific evidence into practical tools and resources to promote healthy eating to Canadians ages 2 years and older. The latest CFG was released in January 2019 and replaced the previous CFG released in 2007 (18). Despite the CFG being recognized as an authoritative guideline for healthy eating, existing research on the 2007 CFG suggests that many Canadians do not use the Food Guide and may not understand it. Although awareness of the 2007 CFG has consistently been reported as being high (19–22), studies suggest that most Canadians do not use the Food Guide as a primary source for healthy eating and nutrition information (20, 21, 23). Furthermore, evidence on the 2007 CFG suggests that many Canadians have a difficult time recalling the four food groups and correctly identifying the number of recommended servings for these food groups based on their age and sex (21, 23–25). A study of the 2007 CFG found that only 43% of adults were able to correctly list all four food groups and <1% were able to recall all food group recommended servings (23).

The 2007 CFG has also received mixed reviews and criticism from researchers, health professionals, and the general public. Specifically, the 2007 CFG has been criticized for being “obesogenic” as it promotes excess calorie consumption and does not take into consideration calories consumed from “other” foods; lacking representation of cultural and traditional foods; being difficult to follow and apply into daily life; and, being highly influenced by the food and beverage industry (19, 22, 26–29).

The 2019 CFG was significantly revised. The rainbow model used in the 2007 CFG was replaced with a plate model. The number of food groups decreased from four to three, with the 2019 CFG amalgamating the Milk & Alternatives and Meat & Alternatives food groups into one Protein Foods food group. The specific recommendations for number of servings per food group based on individual's age and sex in the 2007 CFG was replaced with one universal recommendation for all Canadians based on the proportions of a plate (30, 31). Health Canada also revised the policy process to develop the 2019 CFG by including new rules for advisory committee membership, new and regular evidence review cycles, as well as new stakeholder consultation processes which precluded direct consultation with industry stakeholder and regulated interactions with stakeholders by publishing any communication between Health Canada and stakeholders online (30–33).

Little is known about Canadians' opinions on the 2019 CFG. While a recent survey (34), social media analysis (35), and qualitative study (36) have explored Canadians' opinions of the 2019 CFG, no studies have directly compared opinions of the 2019 to the 2007 CFG. Thus, the objective of this study was to build upon previous research of awareness, use, knowledge, and opinions of CFGs by comparing the perceptions and opinions of the 2007 and 2019 CFGs among parents of young children. Given that parents play a key role in determining their children's eating patterns as well as their own (7), understanding parents' knowledge and perception of food-based dietary guidelines is critical to informing family-based nutrition interventions and policies.

## Materials and methods

### Study design

A multiple cross-sectional study was conducted using data from the Guelph Family Health Study (GFHS), a randomized controlled trial of a family-based intervention focused on improving sleep, screen time, physical activity, and family meal routines among families with preschool aged children. Families were eligible to participate in the GFHS if they had at least one child between 18 months to 5 years, lived in the Guelph area in Ontario, Canada area and had one parent who could respond to questionnaires in English. The data used in this multiple cross-sectional study were drawn from parents who completed a Baseline or 6-month online survey via Qualtrics, between January 2018 to March 2020. From January 2018 to March 2019, the GFHS Baseline and 6-month surveys included questions regarding the 2007 CFG. To reflect Health Canada's revisions to the Food Guide in January 2019, the GFHS surveys were updated in March 2019 to ask parents about their perceptions and opinions of the 2019 CFG. Depending on the timing of families' enrolment in the GFHS, some parents only answered questions about the 2007 CFG (*n* = 250) or the 2019 CFG (*n* = 100), while others (*n* = 77) completed questions about both the 2007 and 2019 CFGs at separate time points. For this study, we examined all parents who completed questions on either the 2007 Food Guide, the 2019 Food Guide, or both the 2007 and 2019 Food Guides, which yielded a total analytic sample of 504 responses. This study was approved by the University of Guelph Research Ethics Board (REB #17-07-003).

### Measures

The survey questions used for this study were drawn from the GFHS Baseline and 6-month survey and were composed of 5 sections: demographics, awareness of CFGs (2 questions), use of CFGs (2 questions), knowledge of CFGs recommendations (11 questions), and opinions of the CFGs (12 questions).

#### Awareness

Parents' awareness of the 2007 CFG was assessed with the question: “Have you heard about Canada's Food Guide?” The questions regarding the 2019 CFG were preceded by a preamble that stated “In **January 2019**, Health Canada released a **new** Canada's Food Guide. Below are some questions to assess your use and opinion about the **new** Canada's Food Guide.” The question to assess parents' awareness of the 2019 CFG asked: “Have you heard about the **new** Canada's Food Guide?” Responses were compared between the two Food Guides and used as a dichotomous measure (yes, I have heard about the Food Guide, or no, I have not heard about the Food Guide).

#### Use

Parents' use of the 2007 and 2019 CFGs was assessed with the question: “What do you use/have you used Canada's Food Guide for?” The question regarding the 2019 CFG asked specifically about using “the **new** Canada's Food Guide.” Parents were provided with several answers and were able to select one or more answers that applied to their family: “to guide my food choices”; “to help me ensure I am feeding my child(ren) healthy foods”; “to plan healthy meals for myself and my family”; “to help me understand portion sizes”; “to help make sure my family and I are getting enough vitamins, minerals, and other nutrients”; “to reduce my risk of chronic diseases such as cancer, diabetes, heart disease”; “to help maintain healthy weights for myself and family”; “to guide my food purchases”; “to help me understand the nutrition facts label”; “to help me limit unhealthy fats, such as saturated fat”; “to help me maintain optimal health and wellness”; “to find healthy recipes; to help me limit salt”; “to help me limit sugar”; “to help me increase plant-based protein”; “to help me limit animal-based protein”; “to help me contribute to the sustainability of the planet”; and, “others: please specify.” Parents' responses to use of CFGs was analyzed using descriptive analysis to provide context to how parents may have used the 2007 and 2019 CFGs.

#### Knowledge

Parents' knowledge of the 2007 and 2019 CFGs were assessed by examining whether parents could correctly identify the recommended number food guide servings for the 2007 CFG and food group proportions for the 2019 CFG. Parents were asked separate questions to assess their knowledge of recommendations for children (2-3 years) and adults (19 to 50 years old, based on parents' reported sex). Responses for parents' knowledge of the 2007 and 2019 CFGs' food group servings and proportions were assessed using a rubric matrix, and answers were coded as correct or incorrect. The total score of parents' knowledge for CFG 2007 food group servings and 2019 proportions for adults and children was calculated by summing the number of correct answers for each food group. Each correct answer was coded as 1 while incorrect answers, “I don't know,” “I am not comfortable answering this question,” or blank answers were coded as 0.

#### Opinions

Using a five point-Likert scale (1 being strongly disagree and 5 being strongly agree), parents' opinions of the 2007 and 2019 CFGs were assessed by asking parents to rank how strongly they agreed or disagreed with the following statements: “Canada's Food Guide is a helpful resource for planning a healthy diet”; “I trust the information provided in Canada's Food Guide”; “I find Canada's Food Guide difficult to understand”; “I find Canada's Food Guide easy to follow”; “I find it difficult to feed my children according to Canada's Food Guide”; and, “Canada's Food Guide is representative of my culture and our traditional foods.” As with the previous questions, the questions regarding the 2019 CFG asked specifically about using “the **new** Canada's Food Guide.” Responses for parents' opinions of the 2007 and 2019 CFG were compared with each statement and used as ordinal measures.

Furthermore, parents were asked to share any other thoughts regarding CFG. These answers were thematically coded to determine common themes among parents' opinions of the 2007 and 2019 CFG; participants provided 126 comments about the 2007 CFG and 47 comments about the 2019 CFG.

### Data analysis

Statistical analysis was conducted using R Statistical Software. To compare the awareness, use, knowledge, and opinions of the 2007 and 2019 CFGs, we compared responses from all parents who completed the questions regarding the 2007 CFG (*n* = 327) and all parents who completed the 2019 CFG questions (*n* = 177) using the following statistical analyses: Pearson's Chi-square for categorical data (e.g., awareness response, knowledge of each CFG food groups serving sizes/plate proportions for adults and children) and Wilcoxon Rank-Sum test for ordinal data (e.g., opinion responses). Descriptive analysis was used to compare and provide context about how parents used the 2007 and 2019 CFG. Thematic coding was used to analyze the responses parent provided to the open-ended questions asking parents to share any additional opinions they may have had about the 2007 and 2019 CFG.

To examine whether participants responses differed by intervention status, we stratified participants based on whether they were randomized into the intervention or control group, and used Pearson's Chi-square and Wilcoxon Rank-Sum tests to compare if any significant differences were observed among awareness, knowledge, and opinions of the 2007 and 2019 CFG. No significant differences were found between the intervention and control groups except for a single item asking whether the 2007 Canada's Food Guide is representative of my culture and our traditional foods (intervention group M = 3.43, control group M = 3.15, *p* = 0.003). Given the lack of substantive differences in responses between participants randomized to intervention and control, we present the unstratified results.

## Results

### Descriptive data

A total of 327 parents and 177 parents answered questions regarding the 2007 and 2019 CFG, respectively. The average age of parents who provided responses regarding the 2007 and 2019 CFGs were 35.8 and 36.6 years, respectively. In both samples, most participants were white, married, highly educated (university education or more), and had relatively high annual household income ($100,000+; Table 1).

**Table 1:** Characteristics of the parents who responded to questions about the 2007 CFG (*n* = 327) and the 2019 CFG (*n* = 177).

**Variables**	**2007** ***n*** **= 327 (%)**	**2019** ***n*** **= 177 (%)**
**Gender**		
Female	195 (59.63)	107 (60.45)
Male	132 (40.37)	68 (38.42)
Gender queer/gender non-conforming	0 (0)	2 (1.13)
**Age, years, Mean (SD)**	35.82 (4.61)	36.59 (4.97)
**Ethnicity**		
White	278 (85.01)	141 (79.66)
Non-White^a^	44 (13.45)	34 (19.20)
Explicitly did not disclose	5 (1.53)	2 (1.13)
**Marital status**		
Married	275 (84.35)	144 (81.35)
Not married, but living with partner	42 (12.88)	21 (11.86)
Single/Separated/Divorced	9 (2.77)	8 (4.50)
**Annual household income, Canadian $**		
< $49,999	30 (9.80)	22 (13.25)
$50,000–$99,999	123 (40.19)	38 (22.89)
$100,000–$149,999	84 (27.45)	63 (37.95)
>$150,000	69 (22.55)	43 (25.90)
**Level of education**		
Some university education or less	97 (29.66)	52 (29.37)
University graduate or more	230 (70.33)	125 (70.62)

a Non-White ethnicity included South Asian (e.g., East Indian, Pakistani, Sri Lankan, etc.), Chinese, Korean, Japanese, Southeast Asian, Black, West Asian, Latin American, and mixed ethnicity.

### Awareness and use

Overall, parents had a high level of awareness of both the 2007 and 2019 CFGs with 94.5% of parents reported having heard of the 2007 CFG, while 90.4% of parents reported having heard of the 2019 CFG. Between the two Food Guides, no significant difference was observed in awareness of the 2007 and 2019 CFGs (*p* = 0.12). Of the parents who were aware of the 2007 and 2019 CFGs and reported using the guides (2007 = 31.39%, 2019 = 51.98%), the top reasons for using both guides were “To help ensure I am feeding my child(ren) healthy meals” and “To guide my food choices” (data not shown).

### Knowledge

**Figure 1:**
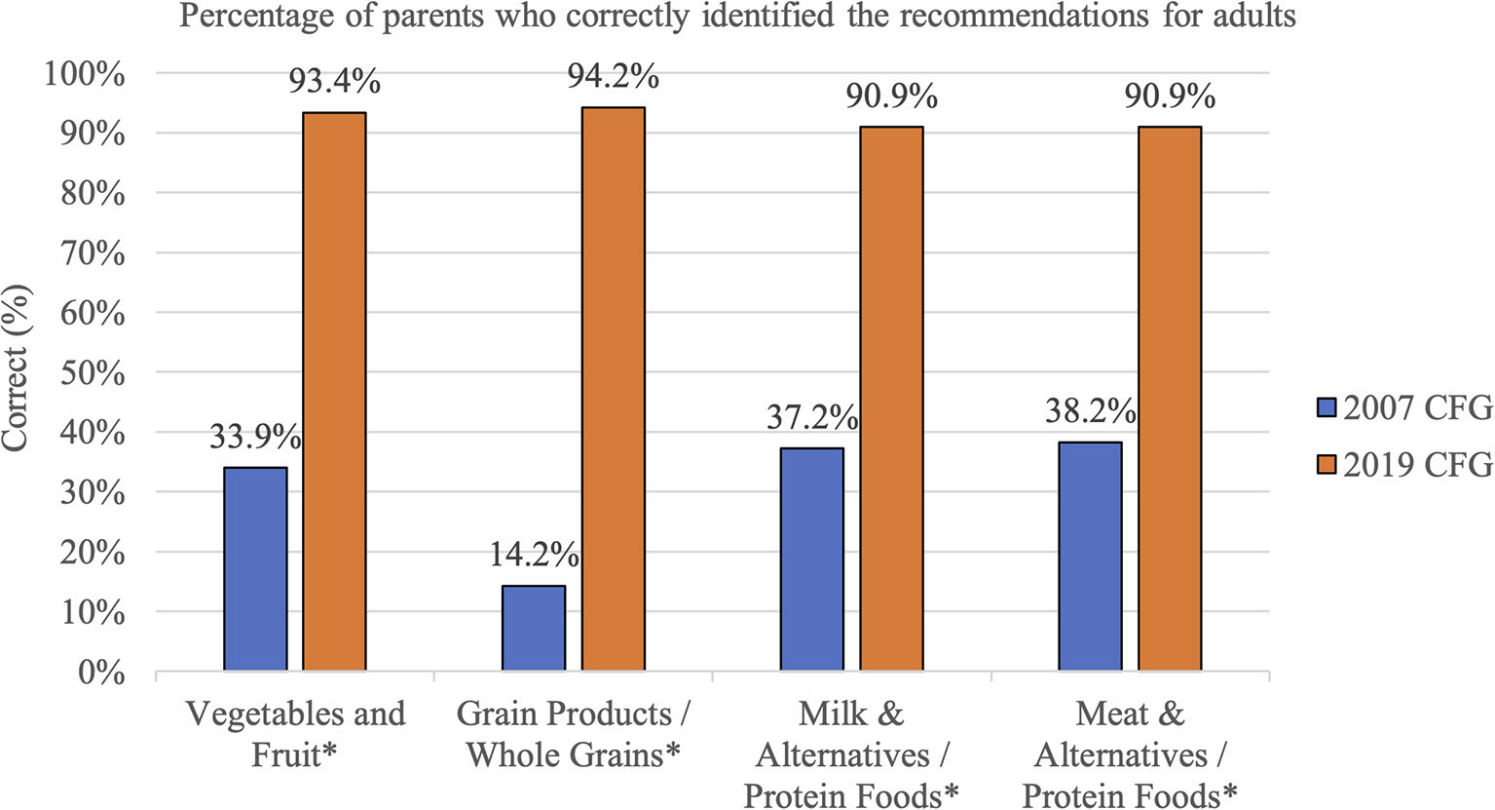
Knowledge of adult CFG food groups serving size (2007) and proportion (2019) recommendations among parents from the GFHS. ^*^Asterisks indicates a statistically significant difference, p < 0.05.

**Figure 2:**
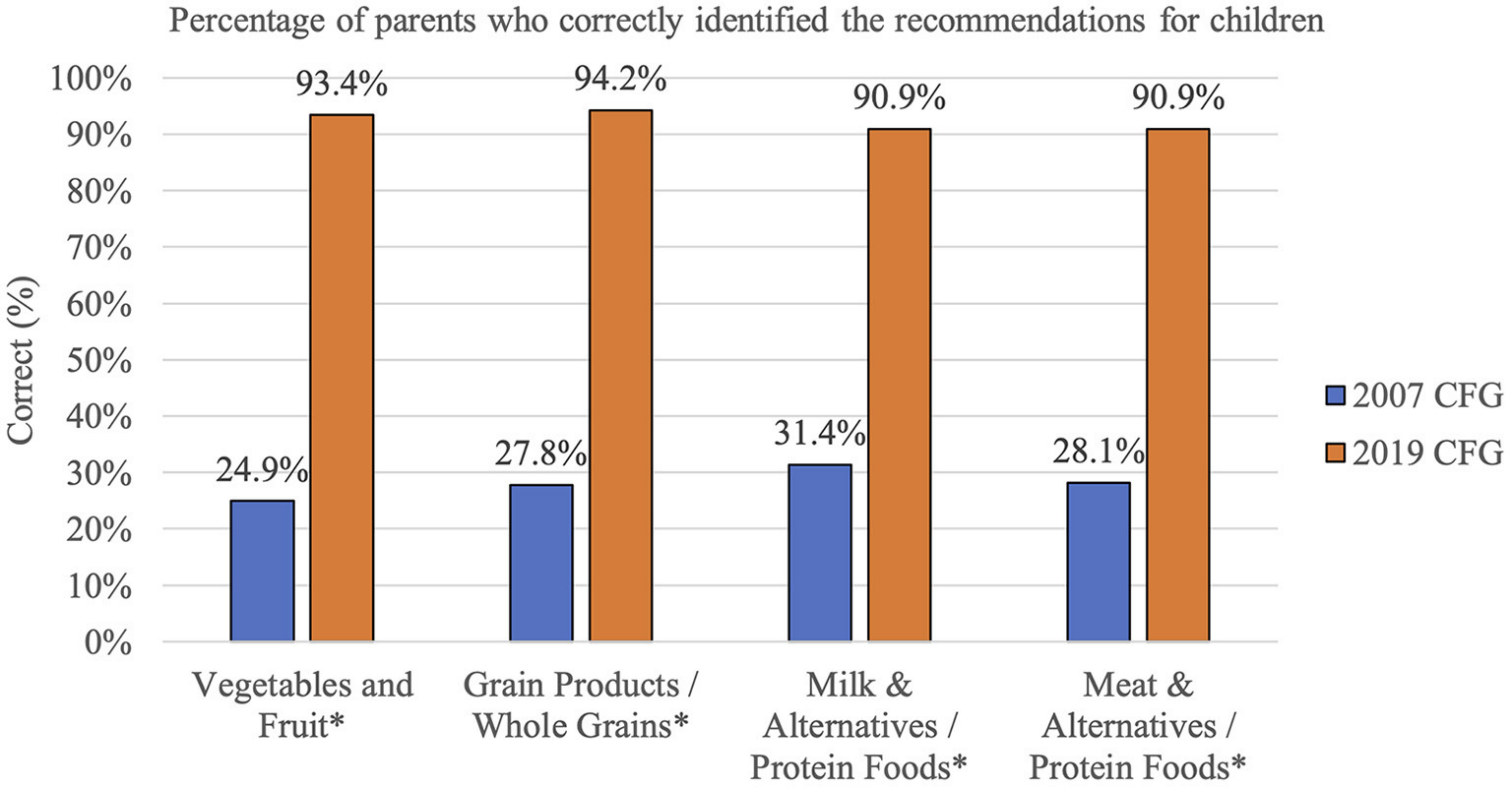
Knowledge of children CFG food groups serving size (2007) and proportion (2019) recommendations among parents from the GFHS. ^*^Asterisks indicates a statistically significant difference, p < 0.05.

Parents' knowledge of the 2019 CFG plate proportion recommendations was higher than their knowledge of the 2007 CFG recommended number of servings. This difference in parents' knowledge was found for both recommendations for adults (19 to 50 years old, based on parents' reported sex) and children (age 2–3 years; Figures 1, 2).

### Opinions

Compared to the 2007 CFG, we found significantly higher mean opinion scores for the 2019 CFG for items assessing whether: (1) the CFG is a helpful resource for planning a healthy diet, (2) parents trust the information provided in the CFG, (3) the Food Guide is easy to follow, and (4) the Food Guide is representative of parents' culture and traditional foods. Compared to the 2007 CFG, mean scores for the 2019 CFG were lower for items assessing if parents find the CFG difficult to understand or if they find it difficult to feed children according to the Food Guide recommendations (Table 2).

**Table 2:** Parents' opinions of the 2007 and 2019 CFGs.

**Opinion**	**2007 CFG *n* = 304^a^**	**2019 CFG *n* = 119^a^**			
	***M***	***M***	***Z***	***p-value***	***Effect size***
Canada's Food Guide is a helpful resource for planning a healthy diet.	3.25	3.78	−5.09	<0.001	0.25
I trust the information provided in Canada's Food Guide.	3.20	4.00	−8.00	<0.001	0.39
I find Canada's Food Guide difficult to understand.	2.31	2.09	−2.33	<0.05	0.12
I find Canada's Food Guide easy to follow.	3.50	3.89	−4.52	<0.001	0.22
I find it difficult to feed my children according to Canada's Food Guide.	2.95	2.44	−5.16	<0.001	0.25
Canada's Food Guide is representative of my culture and our traditional foods.	3.28	3.61	−3.25	<0.001	0.16

1 = Strongly disagree, 2 = Disagree, 3 = Neither agree nor disagree, 4 = Agree, 5 = Strongly Agree.

a Numbers differ slightly due to missing data in the opinion section.

### Themes from open-ended question on the 2007 and 2019 CFG

Common themes that emerged from the open-ended questions included trust of the information provided in the Food Guides, ability to follow and use the Food Guide recommendations, lack of familiarity and use of Food Guide recommendations, and placement of dairy products in the Food Guides.

### Trust of information provided in the food guides

Parents described feelings of distrust with the 2007 CFG, citing industry influence as a major concern: “We find the food company sponsorship in the Canada's Food Guide a conflict of interest, and [it] damages credibility.” Similarly, another parent shared: “I don't pay much attention to [Canada's Food Guide] because my impression is that it's highly influenced by industry lobbying.” Conversely, the 2019 CFG was met with praises and acknowledgment that the foundation of the 2019 CFG was based on current evidence-based information, as one parent wrote: “It is great that they've renewed the Food Guide to better reflect what current research is saying and not just bending to what the heavily subsidized sectors (meat and dairy) want. I'm glad that they've emphasized reducing salt, sugar, and saturated fat, and [focused] on plant-based proteins.”

### Ability to follow and use the food guide recommendations

Regarding comments about the 2007 CFG, parents often reported challenges of conceptualizing and applying the 2007 CFG serving size recommendations: “It is too complicated to remember how many servings of what type and what size each individual member of the family is supposed to aim for.” Similar challenges were also acknowledged by another parent, who wrote: “With all the food that goes unfinished on my child's plate, it's often difficult to track whether portion recommendations are being rigidly met. But we try to keep the ratios balanced throughout the day (2x dairy to meat, 2x produce to dairy, etc.).”

Parents found that the 2019 CFG is easier to follow and more inclusive, as several parents reported: “The proportions of the plate are much easier to understand than portion sizes on the previous food guide…It's visually pleasing,” and “It seems simpler and easier to follow. I'm a vegetarian so it seems more inclusive.” Parents also reported the complementary recipes and resources helped to further their understanding of the 2019 CFG, as one parent described: “It's very user friendly. Easy to read, easy to understand. Great photos to enhance topics. Great recipes and resources.”

### Lack of familiarity and use of food guide recommendations

For both the 2007 and 2019 Food Guides, many parents noted being aware of CFG, however, were not familiar with the specific recommendations. With the 2007 CFG, some parents wrote, “I know of the guide, but I do not know its content” and “I recognize that I think about it in abstract [but] not in details.” Similar comments were also written about the 2019 CFG, as one parent described: “I briefly looked at it when it came out but honestly haven't used it or referenced back to it at all.” Although parents wrote that they were not familiar with the 2019 CFG recommendations, some reported that they still had positive perceptions of the new Food Guide: “I don't recall specifics, but my impression of it was positive,” and “I haven't consulted [the new Food Guide] as I generally think we eat a balanced diet. But I like how it includes things about the food experiences (i.e., eat with others) and the reality of life (i.e., not everyone can cook from scratch all the time, so when at a restaurant, try to make healthier choices).”

While many parents described not consulting the Food Guides, several parents reported “loosely” using the Food Guide recommendations to feed their families: “I don't look at the Guide on a regular basis to plan meals or ‘rate' my family's meals against the serving suggestions included in the Guide; however, I use the principles included in it to guide what my family eats.” Another parent wrote: “I haven't looked at an updated Canada's Food Guide. I would say that I roughly try to make sure we have multiple food groups per meal, including fruit and vegetables at every meal. We don't worry too much about portion sizes for healthy foods – we try to go by hunger/feel.”

### Placement of dairy and dairy products in the food guides

With the 2007 CFG, the Milk & Alternatives food group was often questioned by parents, with some writing: “Too much value placed on milk” and “There [is] a lot of [information] about dairy…not being great for us so I'm curious about [the food group] being on the guide so prominently.” Parents also expressed concerns about family members with milk allergies or lactose intolerance, and not being able to meet the Food Guide recommendations: “…with a 13-month-old with a severe dairy allergy – I find it hard to find milk alternatives that meet the needs to fill that void. Actually, I find it impossible to meet that need.”

One of the most discussed revisions in the 2019 CFG was the amalgamation of the 2007 CFG Milk & Alternatives and Meat & Alternatives food groups into a Proteins Foods food group. Most parents welcomed the change, with many parents supporting the deemphasis of the Milk & Alternatives food group: “It is about time milk was not considered something everyone MUST have,” and “I like the combining of dairy into protein. It is how I already think of it – e.g., I kind of think of the protein food group as a group that includes foods that are high(er) in fat and a source of protein.”

Although most parents agreed that combining the 2007 Food Guide Milk & Alternatives and Meat & Alternatives food groups into one Protein Foods food group in the 2019 CFG was a good idea, some parents expressed concerns about the perceived omission of dairy foods and the potential for inadequate nutrient intake: “I worry about the messages [the Food Guide] sends about calcium consumption (people interpreting it that ‘dairy' is not necessary).” Another parent wrote: “I am concerned that dairy was only listed in protein…I think the new Food Guide should have pointed out that dairy is a good source of protein, and that it should not be cut out in favor of other protein because it is so important for calcium.” One parent also expressed concerns about their child's daycare setting using the new Food Guide recommendations and the influence this may have on their child's diet: “I have concerns that my child's day care…is using the new protein category to take away healthy fats and proteins (such as milk) and provide more plant-based alternatives. The categories are too broad and I'm concerned that they will not provide adequate nutrition for my child.”

## Discussion

This is the first study to compare the knowledge, perceptions, and opinions of the 2007 and 2019 CFGs. Our results revealed that parents had high awareness of both the 2007 and 2019 Food Guides, significantly greater knowledge of the 2019 CFG recommendations, and more positive opinions of the 2019 CFG compared to the 2007 CFG. The exploration of parents' perceptions and opinions of both the 2007 and 2019 CFG is useful to inform our understanding of what parents think and value of food-based dietary guidelines. This understanding is especially important considering that parents' nutrition knowledge and opinions not only influence their own diet and healthy eating habits, but also the growth and development of their young children (7). The results of this study have the potential to advance the thinking about future CFG revisions and inform implementation and knowledge translation strategies for CFG and food-based dietary guidelines in other countries.

The high awareness of the CFGs is consistent with existing research that suggest awareness of CFG to be as high as 80–90% (20, 21, 23, 24, 34). Despite the high level of awareness among participants, studies have found that the use of CFG is low, with Slater and Mudryj's study reporting that only 8.7% of participants had consulted the 2007 Food Guide in the last 6 months (21). Similar findings were noted in our study's quantitative and open-ended responses regarding the 2007 and 2019 CFGs with many parents identifying that they had heard or were aware of the Food Guides, but were not using it to guide their food choices. This observation is not surprising, as participants from Slater and Mudryj's study ranked CFG as the fifth cited source for healthy eating information while participants from Charlebois and colleagues study ranked CFG as the sixth cited source (21, 34). Of the resources consulted for healthy eating information before the Food Guide, participants noted family and friends, general research, TV programs, and social media (21, 34). Research has suggested that consumers value targeted information that is easily accessible, interactive, dynamic, and tailored to their specific needs (37). Given the breadth and complexity of competing healthy eating and nutrition information available, efforts should be put toward creating accessible and tailored healthy eating information, and educating Canadians on how to decipher credible information.

This study also examined parents' understanding of CFG recommendations for adults and children by comparing whether parents could correctly identify the number of Food Guide servings for each food group (Vegetables and Fruit, Grain Products, Milk & Alternatives, and Meat & Alternatives) in the 2007 CFG and the correct plate proportions for each food group (Vegetables and Fruit, Whole Grains, and Protein Foods) in the 2019 CFG. Our study revealed a significant difference among all food groups with parents being able to correctly identify the 2019 plate proportions 91 to 94% of the time. Conversely, only 14 to 38% of parents were able to correctly identify the 2007 CFG food group serving recommendations for adults, with a slightly higher percentage (25 to 31%) of parents correctly identifying 2007 CFG serving recommendations for children. Similar findings about low knowledge of 2007 CFG recommendations have been observed in previous research (21, 23, 24). In Vanderlee and colleagues' study, only 1% of participants could correctly recall the number of servings for all four food groups (23). The low knowledge of the 2007 CFG recommendations are not surprising, as studies examining food guide recommended servings and serving sizes have revealed that they are difficult to conceptualize and apply (22, 36, 38–40).

Across the world, plate models are the second most popular national food-based dietary guideline graphic after the pyramid (41). However, limited research has been conducted to assess the plate model's effectiveness in communicating guidelines. Of the research that has been conducted, the results have suggested that the plate model, which typically includes guidelines for recommended servings of the food groups included on the plate, does not seem to be more effective in communicating healthy eating information compared to other formats like a pyramid or rainbow (42–44). However, research has noted that the plate model is well liked by participants for its visual appeal and modern look (41, 42). It has been suggested that the appeal of a plate model is that it promotes wholeness and a realistic perception of meal planning (41). Unlike hierarchical models, like a pyramid or rainbow, which convey numbers, rankings, and a disjointed, singular view of foods in food groups, the size of each proportion within the plate format dictates their importance to the whole and may resonate with individuals who consume their meals from plates (41). Given this rationale, and the significant increase in parents' ability to recall the 2019 Food Guide guidelines as compared to the food group serving recommendation in the 2007 CFG, future research should explore whether the 2019 CFG plate model translates into improved dietary intake.

Significant differences were observed in the opinions of parents regarding the 2007 and 2019 CFG, with more favorable opinions observed with the 2019 CFG. This finding is consistent with a 2019 survey of Canadian adults, which found that most participants agreed or strongly agreed that the 2019 Food Guide reflected their views and understanding of healthy eating; provided realistic and practical advice; was flexible to meet their dietary preference; was based on scientific evidence and best practices; and could influence food related behaviors (34).

In our study, significantly more parents agreed that they trusted the information in the 2019 CFG as compared to the 2007 CFG. Similar results were observed in the open-answer feedback, where parents cited concerns about the food and beverage industry's involvement in the development of the 2007 Food Guide, whereas parents identified that the 2019 CFG as evidence-based and provided scientifically sound information. These opinions were also noted in a qualitative study among Southwestern Ontario parents, with some parents viewing the 2019 CFG as focusing less on industry interests than previous food guides and acknowledging Health Canada's efforts in creating an evidence-based Food Guide (36). The significant change in opinions of CFG's trustworthy information could be the result of the deliberate effort of Health Canada to work toward more transparent reporting on the CFG development process (30–33). In a study conducted by Weldon and Parkhurst that compared the 2019 CFG to the principles of good governance, it was found that the 2019 Food Guide's development process met 21 out of the 28 measurable indictors of good governance (33). To compare, the authors found that only 6 of the 28 indicators were met by the 2007 CFG (33). Overall, Weldon and Parkhurst concluded that legitimizing good governance like stewardship, transparency, and contestability through the institutionalization of evidentiary processes can help in maintaining public trust of CFG's healthy eating information, which may have significant ramifications for implementing and achieving dietary outcomes (33). To further understand the impact of Health Canada's revised evidence review process for dietary guidance, future research should explore the relationship between Canadians' trust and adherence and use of CFG's healthy eating guidelines.

Parents had conflicting opinions regarding the amalgamation of the 2007 CFG Milk & Alternative and Meat & Alternative food groups into a single Protein Foods food group in the 2019 CFG. Although some parents supported the de-emphasis of the Milk & Alternatives food group in the 2019 Food Guide, some parents expressed concerns about the perceived omission of dairy products and the potential of inadequate intake of nutrients like calcium. Similar results have been shown in previous qualitative studies with Canadian parents (36, 45). A 2022 qualitative study with Canadian parents found that parents expressed concern as to whether dairy and dairy products fit on the 2019 Food Guide, if dairy was still considered “healthy,” and how the perceived omission of dairy would affect their child's growth and development (36). Dairy and dairy products are a good source of nutrients like protein, calcium, vitamin D, phosphorus, and riboflavin, and can positively contribute to children's bone growth and height gain (46, 47). However, evidence from the Canadian Community Health Survey suggest that consumption of fluid milk is declining among all age groups in Canada, and incidences of vitamin D deficiency among Canadian children and adolescents are increasing (3, 48). Researchers have suggested that the decline in dairy intake, and subsequently the rise in calcium and vitamin D deficiency, may be further compounded by not highlighting milk and milk alternatives in the 2019 Food Guide (49). To better understand the impact of amalgamating the 2007 CFG Milk & Alternative and Meat & Alternative food groups, future studies should continue to monitor the intake of dairy and dairy products and calcium and vitamin D intake among Canadians. These results can help inform public health initiatives and educational programs to support correct interpretation of the 2019 CFG, i.e., that dairy products are included on the guide.

Despite our study's strengths, including being the first to compare the perceptions and opinions between two Food Guides, it also has several limitations. Firstly, our study's sample was predominantly homogenous with most participants identifying as white, highly educated, and from households with high annual income >$100,000. Thus, our results may not be generalizable to parents from ethnically diverse backgrounds or low-income households. Future research should examine the perceptions and opinions of CFG from Canadians with diverse socioeconomic and cultural backgrounds by using targeted partner-led recruitment strategies that engage community partners and champions who work with low-income and various cultural communities and by using selective sampling techniques, like quota sampling (50). A second limitation of this study was the timing of the GFHS surveys. Parents were asked about the 2007 CFG from January 2018 to January 2019, nearly 11 to 12 years after the 2007 CFG was initially released. It is unclear whether the timing of the survey played a significant role in participants' recall, knowledge, and opinions of the 2007 CFG's recommendations compared to the 2019 CFG's recommendations. It should also be considered that at the time of updating the GFHS survey with the new 2019 CFG, widespread media coverage of the 2019 CFG was present, with traditional media outlets and social media platforms frequently reporting on the updated recommendations, and interviewing nutrition and health professionals on their perspectives and opinions. It is unclear how often participants were exposed to these frequent media messages, and whether they had a significant influence in shaping parents' awareness, use, knowledge, and opinions of the 2019 CFG.

Our study results have implications for future policies and knowledge translation efforts regarding food-based dietary guidelines. Our results suggest that parents value the emphasis of current scientific evidence in developing the 2019 CFG and appear to have improved trust in healthy eating guidelines when industry involvement is regulated. Thus, to continue with public trust, Health Canada and other public health agencies should continue to implement transparent processes that safeguard the credibility of healthy eating guidelines. Second, parents acknowledged that the plate model was easier to follow, and that the complementary resources and recipes aided in their understanding of the Food Guide guidelines. Therefore, future Food Guide revisions should consider knowledge translation strategies that support the practical application of the Food Guide recommendations, and more specifically, that these strategies are culturally and contextually appropriate. Lastly, parents conflicting opinions on the amalgamation of the 2007 CFG Milk & Alternative and Meat & Alternative food groups, and confusion on where dairy and dairy products fit in the 2019 CFG may indicate that a knowledge gap exists among parents about the 2019 CFG Protein Foods food group. Future Food Guide revisions should consider providing clearer messages on the importance of consuming a variety of protein-rich foods, including dairy, and provide more guidance on the consumption of milk and milk alternatives for families with young children.

Overall, our study found that parents in the GFHS had a high level of awareness of both the 2007 and 2019 CFGs, greater knowledge of 2019 CFG plate proportion recommendations, and more positive opinions of the 2019 CFG compared to the 2007 CFG. Specifically, parents felt that the new Food Guide was a helpful and trustworthy resource, easier to follow and understand, and representative of their culture and traditional foods. Future studies examining Canadians' perceptions and opinions of CFG should be conducted among more culturally and socioeconomically diverse samples to allow for more generalizable results. Future research should also investigate whether awareness and knowledge of the 2019 CFG is associated with improved adherence to the 2019 CFG plate proportions and key healthy eating guidance.

## Data availability statement

The raw data supporting the conclusions of this article will be made available by the authors, without undue reservation.

## Ethics statement

The studies involving human participants were reviewed and approved by University of Guelph Research Ethics Board (REB #17-07-003). The patients/participants provided their written informed consent to participate in this study.

## Author contributions

JH, DM, AD, and LF: conception and design of the research. AR and AS: statistical analysis. JH and DM: obtained funding. AR: writing of the manuscript. All authors were involved in the interpretation of the data, critical revision of the manuscript, and also read and approved the final draft.

## Funding

This research was supported by funding from the Canadian Institutes for Health Research and The Helderleigh Foundation.

## Conflict of interest

The authors declare that the research was conducted in the absence of any commercial or financial relationships that could be construed as a potential conflict of interest.

## Publisher's note

All claims expressed in this article are solely those of the authors and do not necessarily represent those of their affiliated organizations, or those of the publisher, the editors and the reviewers. Any product that may be evaluated in this article, or claim that may be made by its manufacturer, is not guaranteed or endorsed by the publisher.
