# Supplementary material for: Comparing the perceptions and opinions of the 2007 and 2019 Canada's food guides among parents of young children
Source: Front Public Health. 2022 Aug 9;10:944648. doi: 10.3389/fpubh.2022.944648 (PMC9395618; doi:10.3389/fpubh.2022.944648)
Supplement: Supplementary file 1 [file Data_Sheet_1.docx]

**Supplementary Figure 1.** Rubric matrices to assess GFHS parents’ knowledge of CFGs adult’s recommendations (based on participants’ sex).

| **2007 Canada’s Food Guide** | | | **2019 Canada’s Food Guide** | | | |
| --- | --- | --- | --- | --- | --- | --- |
| **Food Groups** | **Correctly Identified**  **Number of Servings** | | **Food Groups** | **Correctly Identified**  **Proportions** | |  |
| Vegetables & Fruit | Dichotomous | | Vegetables &  Fruit | Dichotomous | |  |
|  | Males | |  | CORRECT  (50%) | INCORRECT  (10%, 25%, 75%) |  |
|  | CORRECT  (*Any answer: 8 servings, 9 servings, 10 servings) | INCORRECT  (1 serving, 2 servings, 3 servings, 4 servings, 5 servings, 6 servings, 7 servings, 11 servings, 12 servings, I don’t know) |  |  |  |  |
|  | Females | |  |  |  |  |
|  | CORRECT  (*Any answer: 7 servings, 8 servings) | INCORRECT  (1 serving, 2 servings, 3 servings, 4 servings, 5 servings, 6 servings, 9 servings, 10 servings, 11 servings, 12 servings, I don’t know) |  |  |  |  |
| Grain Products | Dichotomous | | Whole Grains | Dichotomous | |  |
|  | Males | |  | CORRECT  (25%) | INCORRECT  (10%, 50%, 75%) |  |
|  | CORRECT  (8 servings) | INCORRECT  (1 serving, 2 servings, 3 servings, 4 servings, 5 servings, 6 servings, 7 servings, 9 servings, 10 servings, 11 servings, 12 servings, I don’t know) |  |  |  |  |
|  | Females | |  |  |  |  |
|  | CORRECT  (*Any answer: 6 servings, 7 servings) | INCORRECT  (1 serving, 2 servings, 3 servings, 4 servings, 5 servings, 8 servings, 9 servings, 10 servings, 11 servings, 12 servings, I don’t know) |  |  |  |  |
| Milk & Alternatives | Dichotomous | | Protein Foods | Dichotomous | |  |
|  | Males | |  | CORRECT  (25%) | INCORRECT  (10%, 50%, 75%) |  |
|  | CORRECT  (2 servings) | INCORRECT  (1 serving, 3 servings, 4 servings, 5 servings, 6 servings, 7 servings, 8 servings, 9 servings, 10 servings, 11 servings, 12 servings, I don’t know) |  |  |  |  |
|  | Females | |  |  |  |  |
|  | CORRECT  (2 servings) | INCORRECT  (1 serving, 3 servings, 4 servings, 5 servings, 6 servings, 7 servings, 8 servings, 9 servings, 10 servings, 11 servings, 12 servings, I don’t know) |  |  |  |  |
| Meat & Alternatives | Dichotomous | |  |  | |  |
|  | Males | |  |  |  |  |
|  | CORRECT  (3 servings) | INCORRECT  (1 serving, 2 servings, 4 servings, 5 servings, 6 servings, 7 servings, 8 servings, 9 servings, 10 servings, 11 servings, 12 servings, I don’t know) |  |  |  |  |
|  | Females | |  |  |  |  |
|  | CORRECT  (2 servings) | INCORRECT  (1 serving, 3 servings, 4 servings, 5 servings, 6 servings, 7 servings, 8 servings, 9 servings, 10 servings, 11 servings, 12 servings, I don’t know) |  |  |  |  |

**Supplementary Figure 2.** Rubric matrices to assess GFHS parents’ knowledge of CFGs children’s recommendations.

| **2007 Canada’s Food Guide** | | | **2019 Canada’s Food Guide** | | | |
| --- | --- | --- | --- | --- | --- | --- |
| **Food Groups** | **Correctly Identified Number of Servings** | | **Food Groups** | **Correctly Identified Proportions** | |  |
| Vegetables &  Fruit | Dichotomous | | Vegetables &  Fruit | Dichotomous | |  |
|  | CORRECT  (4 servings) | INCORRECT (1 serving, 2 servings, 3 servings, 5 servings, 6 servings, 7 servings, 8 servings, 9 servings, 10 servings, I don’t know) |  | CORRECT  (50%) | INCORRECT  (10%, 25%, 75%) |  |
| Grain Products | Dichotomous | | Whole Grains | Dichotomous | |  |
|  | CORRECT  (3 servings) | INCORRECT (1 serving, 2 servings, 4 servings, 5 servings, 6 servings, 7 servings, 8 servings, 9 servings, 10 servings, I don’t know) |  | CORRECT (25%) | INCORRECT  (10%, 50%, 75%) |  |
| Milk & Alternatives | Dichotomous | | Protein Foods | Dichotomous | |  |
|  | CORRECT (2 servings) | INCORRECT (1 serving, 3 servings, 4 servings, 5 servings, 6 servings, 7 servings, 8 servings, 9 servings, 10 servings, I don’t know) |  | CORRECT  (25%) | INCORRECT  (10%, 50%, 75%) |  |
| Meat & Alternatives | Dichotomous | |  | | | |
|  | CORRECT  (1 servings) | INCORRECT (2 servings, 3 servings, 4 servings, 5 servings, 6 servings, 7 servings, 8 servings, 9 servings, 10 servings, I don’t know) |  | | | |
